# Assessing the suitability of fused deposition modeling to produce acrylic removable denture bases

**DOI:** 10.1002/cre2.880

**Published:** 2024-05-27

**Authors:** Khalid K. Alanazi, Duncan Wood, Joanna Shepherd, Christopher W. Stokes, Ilida Ortega Asencio

**Affiliations:** ^1^ School of Clinical Dentistry University of Sheffield Sheffield UK; ^2^ Conservative Dental Science Department, College of Dentistry Prince Sattam Bin Abdulaziz University Saudi Arabia

**Keywords:** 3D‐printed denture, fused deposition modeling (FDM), polymethylmethacrylate (PMMA), stereolithography (SLA)

## Abstract

**Objective:**

To study the feasibility of using poly methyl methacrylate (PMMA) filament and fused deposition modeling (FDM) to manufacture denture bases via the development of a study that considers both conventional and additive‐based manufacturing techniques.

**Materials and Methods:**

Five sample groups were compared: heat and cold cured acrylic resins, CAD/CAM milled PMMA, 3D‐printed PMMA (via FDM), and 3D‐printed methacrylate resin (via stereolithography, SLA). All groups were subjected to mechanical testing (flexural strength, impact strength, and hardness), water sorption and solubility tests, a tooth bonding test, microbiological assessment, and accuracy of fit measurements. The performance of sample groups was referred to ISO 20795‐1 and ISO/TS 19736. The data was analyzed using one‐way ANOVA.

**Results:**

Samples manufactured using FDM performed within ISO specifications for mechanical testing, water sorption, and solubility tests. However, the FDM group failed to achieve the ISO requirements for the tooth bonding test. FDM samples presented a rough surface finish which could ultimately encourage an undesirable high level of microbial adhesion. For accuracy of fit, FDM samples showed a lower degree of accuracy than existing materials.

**Conclusions:**

Although FDM samples were a cost‐effective option and were able to be quickly manufactured in a reproducible manner, the results demonstrated that current recommended testing regimes for conventionally manufactured denture‐based polymers are not directly applicable to additive‐manufactured denture base polymers. Therefore, new standards should be developed to ensure the correct implementation of additive manufacturing techniques within denture‐based fabrication workflow.

## 1 INTRODUCTION

Denture base materials should present key properties to successfully perform in the patients' mouth; these include appropriate esthetics, thermal conductivity, biocompatibility to prevent any adverse reaction with oral tissues and an adequate bond to artificial teeth. Moreover, denture base materials should be able to be polished to a smooth finish with low surface roughness to prevent the adhesion of microbial films. These materials must also have appropriate mechanical properties to deal with high forces generated during function by the occlusion (Messersmith et al., 1998; Zarb et al., 2013) and in service by the user (accidental dropping, brushing, and cleaning). Finally, denture base materials should demonstrate low water sorption and solubility rates since high rates of these parameters can lead to weakening of their mechanical performance as well as altering their dimensional stability, issues that can result in the irritation of the oral soft tissues (Anusavice et al., 2012; Cucci et al., 1998; Dhir et al., 2007; Pfeiffer & Rosenbauer, 2004).

Poly methyl methacrylate (PMMA) acrylic resin is the most frequently used material for the fabrication of denture bases and this is because of its excellent appearance, ease of manipulation, and simplicity of repair, despite not presenting high thermal conductivity (Anusavice et al., 2012). Acrylic PMMA dentures are usually fabricated by compression modeling (flasking and packing); however, this technique presents clear limitations. Processing dentures through this method may take up to 2 days, making it a time‐consuming laboratory procedure that may not deliver a construct with the required optimum mechanical properties and may result in altered dimensionality due to polymerization shrinkage and molding discrepancies (Gharechahi et al., 2014).

With the introduction of computer‐aided manufacturing (CAM), there are now feasible alternatives to conventional flasking and packing. Potential cost savings can be achieved through CAM approaches in dentistry as a considerable amount of costs can be significantly reduced (Van Noort, 2012); these include, for example, the working time of highly skilled technicians as well as materials consumption. Milling systems (subtractive manufacturing) can now be used to create denture base materials via the utilization of pre‐polymerized PMMA blocks; these dentures have been shown to be dimensionally accurate, have good strength, and decreased bacterial adhesion (Miyazaki et al., 2009). Despite these advantages, subtractive manufacturing has been described as overly wasteful, as much of the material that is removed from blocks to produce the final form is discarded (Van Noort, 2012). In addition, milling burs wear quickly and thus demand frequent replacement (Saratti et al., 2019).

Additive manufacturing (3D printing) approaches are a potential solution to overcome some of the issues presented by conventional and subtractive techniques. Prostheses such as crowns, implants, and bridges are examples of dental devices that are currently being successfully fabricated by additive manufacturing (Bogue, 2013). The most used additive techniques are stereolithography (SLA), selective laser sintering (SLS), and fused deposition modeling (FDM) (Gaal et al., 2017). In SLA, a power source, such as UV, is used to cure photocurable resins layer by layer, while in SLS technology, a laser is used to combine small pieces of a material powder into an object with the required 3D structure. The FDM technique follows an extrusion principle where a molten thermoplastic material is extruded from the head of a printer on a mobile platform (Van Wijk & van Wijk, 2015); this technique offers a more cost‐effective approach as it can make use of newly developed 3D‐printing filaments and low‐cost 3D printers. There are currently many unknown variables related to the FDM method, such as optimizing the print settings for acceptable mechanical properties as well as the accuracy and surface finish of the final prints.

With the increasing sophistication of 3D printing techniques in dental applications, it was timely to evaluate the possibility of using FDM 3D printing for denture base manufacturing. Therefore, the purpose of this study was to evaluate FDM against subtractive milling and conventional methods for acrylic denture base fabrication and assess its effectiveness with respect to a series of key properties. These properties included mechanical performance, water sorption, and solubility, tooth bonding, surface adherence for microbes, and accuracy of fit (following specific denture base testing ISO standards). Our null hypothesis is that denture‐based manufacturing techniques would have no effect on the key selected properties.

## 2 MATERIALS AND METHODS

**Figure 1:**
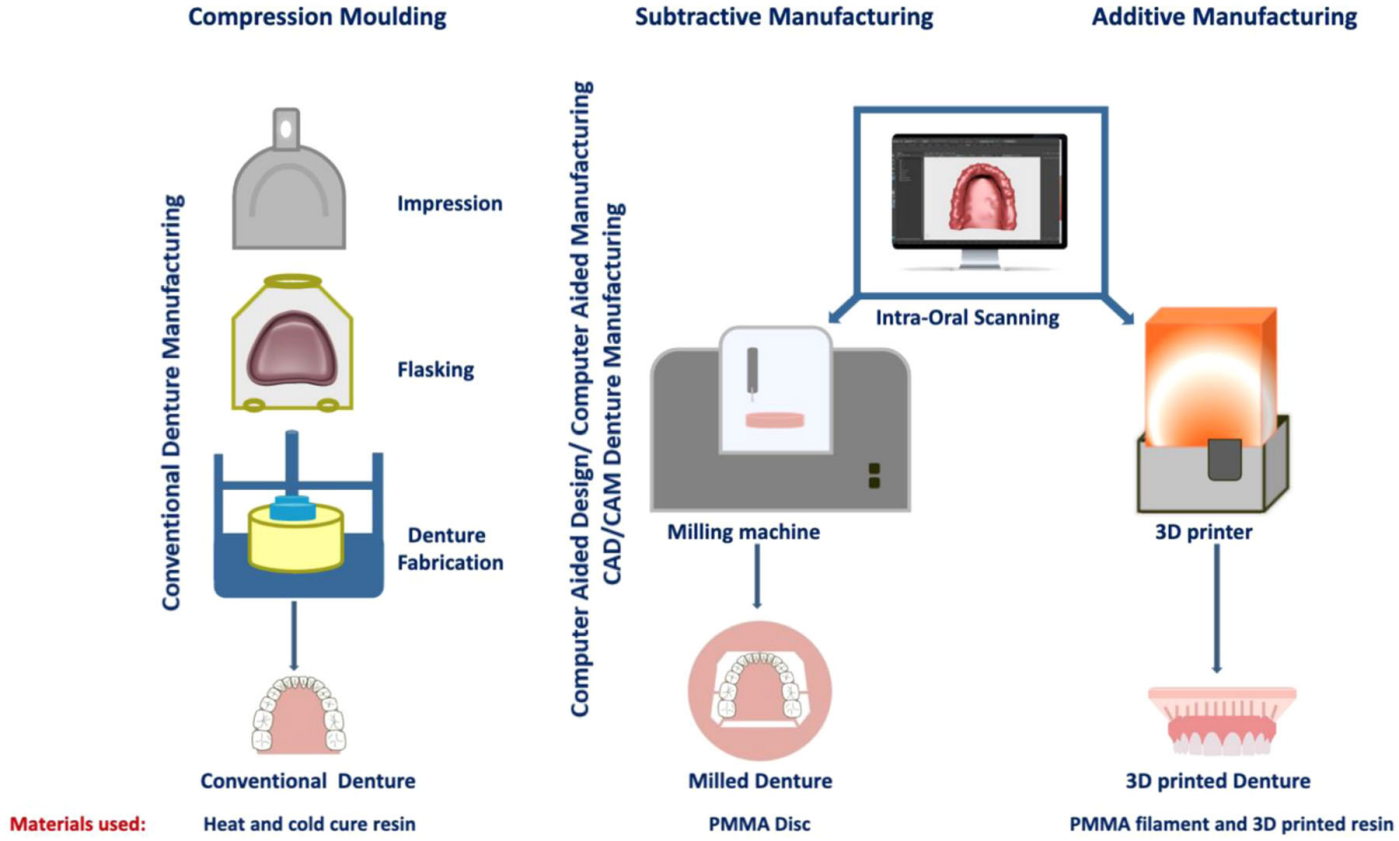
Schematic chart of denture fabrication strategies, including conventional and digital manufacturing approaches (with materials used for each technique). Conventional denture manufacturing by alginate impression and flasking (compression molding). Digital denture manufacturing using an intraoral scanner (digital impression); fabrication of denture either by subtractive technique (milling) or additive technique (3D printing).

PMMA samples were distributed between five groups: (1) heat cured, (2) cold cured, (3) milling (control groups), (4) additive (FDM), and (5) additive (SLA) (experimental groups). A list of the materials, manufacturers, composition, and denture base fabrication techniques is illustrated in Table 1. The process of fabrication of the denture via conventional and CAD/CAM techniques is shown in Figure 1. Also, 3D‐printed samples were printed in three orientations: X, Y, and Z for mechanical tests and two orientations (X and Y) for the rest of the tests. This was done to test for variabilities in properties caused by the layer‐by‐layer manufacturing process.

**Table 1:** Materials, manufacturers, composition, and denture base fabrication techniques.

Material	Manufacturer	Composition	Fabrication technique
Group 1 Probase hot	Ivoclar Vivadent AG	Powder: PMMA, benzoyl peroxide, pigments, plasticizer Liquid: 85%−95% MMA, 5%−10% EGDMA, catalysts	Conventional (compression)
Group 2 Probase cold	Ivoclar Vivadent AG	Powder: PMMA, benzoyl peroxide, pigments, plasticizer liquid: 90%−95% MMA, <5% butandiole, dimethacrylate, catalysts	Conventional (compression)
Group 3 IvoBase CAD	Ivoclar Vivadent AG	PMMA	Subtractive
Group 4 3D filament	Material4print	PMMA	Additive (FDM)
Group 5 Denture base resin	Formlabs Inc.	Methacrylate monomer, diurethane dimethacrylate, propylidynetrimethyl trimethacrylate	Additive (SLA)

Abbreviations: CAD, computer‐aided design; EGDMA, ethylene glycol dimethacrylate; FDM, fused deposition modeling; MMA, methyl methacrylate; PMMA, polymethylmethacrylate; SLA, stereolithography.

### 2.1 Manufacturing of samples

Flexural samples' shape, dimension, and number were determined according to the ISO 20795‐1:2013 specification for denture base polymers (International Organization for Standardization, 2013).

Heat‐cured samples (Group 1): Samples (*n* = 5) were prepared using a plaster mold made by investing rectangular shaped wax patterns (64 × 10 × 3.3 mm) then the wax was removed after plaster setting, the acrylic dough ProBase® Hot (Ivoclar Vivadent AG) was mixed and packed into the plaster molds, according to the manufacturer's instructions. The material was cured for 6 h at 95° C. The rectangular resin samples were then removed from the flask.

Cold‐cured samples (Group 2): (*n* = 5) samples with dimensions (64 × 10 × 3.3 mm) were prepared by mixing powder and liquid of cold cure acrylic resin ProBase® Cold (Ivoclar Vivadent AG), according to the manufacturer's instructions. The resin was poured into the custom mold and placed in a pressure polymerization unit (Polymax 5; Dreve) for 15 min at 40°C. After bench cooling, the acrylic samples were removed from the mold.

Milling samples (Group 3): A design with the dimensions (64 × 10 × 3.3 mm) using Fusion 360™ software (Autodesk Inc.) was created and then imported into SUM3D to configure the milling parameters Five samples were milled with a 5‐axis milling machine (Roland DWX‐50) from a PMMA disc (IvoBase CAD).

Additive (FDM) (Group 4): The same design used in Group 3 was imported into Cura (LulzBot Edition version 2.6.52), which was used to set up the printing parameters. Five samples for each XY printing position were printed using a desktop 3D printer (LulzBot TAZ 6; Aleph Objects Inc.), using a 3D filament of PMMA (Material4print), and using the manufacturer's instructions for building the plate temperature 100°C and 250°C for the printing temperature. The diameter of this filament was 2.85 mm, and the infill density of printing was set to 100%.

Additive (SLA) (Group 5): The same design used in Group 3 was imported into PreForm Software (version 2.18.0), which was used to set up the printing parameters. This group has two subgroups since two different resins (gray resin and denture base resin; Formlabs Inc.) were used. For each subgroup, five samples for each XY printing position were printed using a desktop 3D printer (Form 2; Formlabs Inc.).

Following the manufacturing process, flexural samples were finished by using wet grinding with P500, 1000, and 1200 grit paper (SiC grinding paper; Buehler) and stored in water at 37°C for 50 ± 2 h before the flexural test according to the ISO 20795‐1:2013 specification (International Organization for Standardization, 2013).

The impact samples' shape, dimension, and number were determined according to the ISO 20795‐1:2013 specification for denture base polymers (International Organization for Standardization, 2013). All samples (*n* = 10) for each group were produced using the same method as previously described in Section 2.1.1 but with dimensions of 39 × 8 × 4 mm. Following production, all samples were subjected to motorized notch cutting (RAY‐RAN TEST EQUIPMENT LTD) to create a “v”‐shaped notch at the center of each impact sample according to the ISO 20795‐1:2013 specification for denture base polymers. The impact samples were finished using the same procedure of finishing flexural samples and stored in water at 37°C for 7 days ±2 h before impact test according to the ISO 20795‐1:2013 specification (International Organization for Standardization, 2013). The remaining samples of all groups that were prepared for the impact test were used as hardness samples; two samples with five readings on each sample. Additionally, these samples were subjected to mechanical polishing with polishing paste using a dental lathe (wet pumice on a rag wheel) and then polished with an ultra‐shine rag wheel to mimic the conventional method of polishing an acrylic denture base. All these procedures were performed by one investigator.

The water sorption and solubility samples' shape, dimension, and quantity were determined according to the ISO 20795‐1:2013 specification for denture base polymers (International Organization for Standardization, 2013). All samples (*n* = 5) for each group were produced using the same method as previously described in Section 2.1.1 but with the disc‐shaped dimensions of 50 × 1 mm. 3D‐printed samples were printed in two orientations: X and Y. Following manufacturing, all group samples were finished by using wet grinding with P500, 1000, and 1200 grit paper (SiC grinding paper; Buehler) to reach the desired thickness of (0.5 ± 0.1) mm according to the ISO specifications (International Organization for Standardization, 2013).

The tooth bonding samples' shape, dimension, and number were determined according to ISO specifications of the tooth bonding test (ISO/TS, 2017) (International Organization for Standardization, 2017). Tooth bonding samples (*n* = 6) for each group were produced using the method described in Section 2.1.1. In addition to this, a “practice‐based” (not ISO specifications) method of sample fabrication was performed on heat cure, cold cure, milling, and the X SLA denture resin groups. This method includes roughening the fitting surface of prefabricated teeth using a micromotor to remove the glaze layer and then painting methyl methacrylate monomer on the prepared surface. Also, X SLA denture resin groups were prepared using uncured denture base resin (A) and self‐cure (ortho resin) (B) as bonding agents.

A sample shape with dimensions 10 mm diameter and 2 mm thickness was used for the assessment of microbial adherence, including surface roughness, wettability, viability test, and scanning electron microscopy (SEM). Samples were produced for each group using the method described in Section 2.1.1. Following manufacturing of the samples, each sample group was divided into (A) as processed and (B) standardized finishing surface by using wet grinding with P600 Grit paper (SiC grinding paper; Buehler) on each surface five times; then, samples were rotated around 90° and ground another five times. In addition to this, a further group of the FDM group was subjected to acetone vapor finishing, which was performed in a glass desiccator containing acetone with the samples kept inside the desiccator for 2 h.

A cast of an edentulous maxillary arch with three spherical reference points was defined as a master cast. The three spheres were used to standardize the digital process of alignment between the cast and the denture base through the various samples and groups. The master cast was duplicated using silicone‐based duplication material (FINOSIL 15 Duplicating Silicone; A and B; FINO) according to the manufacturer's instructions to produce a master silicone mold. This mold was used to fabricate the experimental model. Six experimental models were assigned for each heat and cold cure group, and one experimental model was assigned for CAD/CAM groups (milling, FDM, and SLA). SLA and FDM denture bases were printed in two orientations. For the fabrication of experimental denture bases of each group, the method used in Section 2.1.1 was followed.

### 2.2 Characterization/testing of samples

#### 2.2.1 Flexural strength test

Flexural samples were subjected to the three‐point flexural strength test according to the ISO 20795‐1:2013 using a universal testing machine (Lloyd LRX; AMETEK Inc.). Samples were centrally located and a load of 2.5 kg at a crosshead speed of 5 mm/min was applied until fracture occurred. The span length was 50 mm. Computer software (NEXYGEN 4.1; Lloyd Instruments) was used to obtain the flexural strength value for each group.

#### 2.2.2 Impact strength test

Impact samples were subjected to a Charpy impact test using an impact tester (H503 Impact test; Tinius Olsen Ltd). The samples were positioned centrally with the V notch facing the opposite side of the pendulum of the testing machine. With the pendulum released from its rest position, the sample was fractured, and the maximum load before fracture was recorded as the impact strength in joules.

#### 2.2.3 Vickers hardness test

Hardness was measured using a Vickers hardness tester (Foundrax) with an applied load of 1 kg for a 10 s dwell time. The diagonal lengths (D1 and D2) of a square shape trace were measured by using a scaled microscope. The mean value of five points of indentation for each hardness sample was used to obtain the Vickers hardness.

#### 2.2.4 Water sorption and solubility tests

The process of the test was according to ISO specifications (International Organization for Standardization, 2013) and consisted of three steps.

#### 2.2.5 Conditioned samples

Each group of samples was placed in a rack inside a desiccator containing dried silica gel. The desiccator was kept in the oven at 37°C for 24 h followed by the transfer of samples to the second desiccator supplied with new silica gel. The second desiccator was kept at 24°C for 1 h then the samples were weighed using an analytical weighing balance (Mettler; AJ‐100). The desiccator remained closed throughout the process except for removing and replacing samples. After weighing all samples, the silica gel in the first desiccator was replaced. The cycle described above was repeated until obtaining a constant mass, called conditioned mass m1, where the loss in mass of each sample is not more than 0.2 mg between two consecutive measurements. After that, the volume (mm^3^) of each sample was measured by calculating the mean of three diameter measurements and the mean of five thickness measurements at four equally spaced locations at the circumference of the sample, together with a center measurement.

#### 2.2.6 Wet samples

The samples were immersed in a water bath (VWR Collection) at 37°C for 7 days. The samples were then removed from the water, wiped with a clean, dry towel, and allowed to air dry for 15 s. They were then weighed after 60 s of removal from the water, and each sample's weight was recorded as wet mass, m2.

#### 2.2.7 Reconditioned samples

The samples were reconditioned to constant mass with the same conditions applied to the first drying process as described early in the first step. The samples' weight was recorded as reconditioned mass, m3.

Water sorption, Wsp, value can be calculated by applying the following equation:

Wsp=m2−m3/v

Water solubility, Wsl, value can be calculated by applying the following equation:

Wsl=m1−m3/v

m1 is the conditioned mass of the sample in μg,

m2 is the wet mass of the sample in μg,

m3 is the reconditioned mass of the sample in μg,

V is the volume of the sample in mm.^3^

### 2.3 Tooth bonding test

Tooth bonding samples were subjected to a shear strength test, according to ISO/TS 19736: 2017 (International Organization for Standardization, 2017), using a Lloyd LRX Universal Testing Machine (AMETEK Inc.). A vertical load of 2.5 kg with a speed of 1 mm/min was applied by the shear pin on the incisal edge of the palatal surface of the artificial tooth until a fracture occurred. Then shear strength values were obtained from computer software (NEXYGEN 4.1; Lloyd Instruments) connected to the testing machine. Also, the mode of fracture (adhesive, cohesive, or mixed) was visually examined.

#### 2.3.1 Microbiological adherence assessment

Surface roughness was measured using a profilometric device (TR200; Time Group Inc.) in conjunction with a 0.2 μm diamond tip. This profilometer was set to move the diamond sensor across the sample surface with 1.25 mm. Three samples of each sample group for each surface condition were subjected to three readings in three different directions (oblique, transverse, and linear) to measure any expected surface irregularities. The surface roughness (Ra) values were calculated in microns, and all measurements were performed by one operator.

Surface wettability is determined by measuring the contact angles between the distilled water drop and the sample's surface. Contact angles were measured by sessile drop method using a Drop Shape Analyzer device (DSA100; KRÜSS). Three samples from each group with different surface conditions were subjected to two distilled water drops (each 5 μL) in different areas of each sample. Then the mean of the right and left contact angle of each drop was calculated.

#### 2.3.2 Fungal growth

Biofilms of *Candida albicans*, a common oral fungal commensal and opportunistic pathogen, were grown on the surface of three samples of A, B, and C from each group, and on a glass disc inside the well plate (TCP) as a positive control. Uninoculated samples were used as a negative control. The experiment was repeated three times as follows:

Stock plates of *C. albicans* strain BWP17 on YPD (yeast, peptone, and dextrose) agar were used. A single colony of *C. albicans* BWP17 was transferred to 15 mL YPD broth and incubated overnight at 37°C. Three samples of each group were sterilized by immersion in industrial methylated spirits for 15 min. The samples were then transferred to a new 24‐well plate and washed with 1 mL of phosphate‐buffered saline (PBS). *C. albicans* from overnight cultures were diluted in YPD broth to a concentration of 7.5 × 10^5^ colony forming units (CFU) per mL, established using the Miles and Misra viable counting method alongside an optical absorbance reading. One milliliter of YPD broth was added to each sample, including the positive and negative controls. Ten microliters of *C. albicans* suspension containing ~7.5 × 10^3^ cells was added to each well with the exception of the negative controls. The plates were then incubated at 37°C for 24 h, following which metabolic analysis was performed.

All samples were transferred to a new 24‐well plate and were washed with PBS. Five hundred microliters of 10% Presto Blue was added to each well, and plates were incubated at 37°C for 1 h. After incubation, 200 μL of the solution from each well was transferred to a 96‐well plate in duplicate, and the absorbance was measured using a spectrophotometer Infinite 200 PRO Microplate Reader with an excitation wavelength of 550 nm and an emission wavelength of 590 nm (TECAN).

### 2.4 SEM of adherent *Candida*

SEM was also used to analyze early *Candida* biofilm formation in sample groups. Samples were washed twice with PBS then fixed with 2.5% glutaraldehyde and 0.1 M sodium cacodylate buffer overnight, then washed three times with PBS and one time with distilled water. Following dehydration in a graded series of ethanol solutions, samples were dried by a 1:1 mixture of hexamethyldisilazane (HMDS) and 100% ethanol and then dried finally in 100% HMDS. Following that, HMDS was removed, and samples were left to dry in a fume hood. Samples were fixed onto the pin specimen holder and then gold coated by a sputter coater unit (Edwards S150B). Samples were examined by using a Scanning Electron Microscope (Tescan Vega3 LMU) with an operating voltage of 15 kv.

#### 2.4.1 Accuracy of fit study

For conventional groups, each scan of the denture base was registered/aligned and compared with their corresponding scan of the experimental model by using matching software (CloudCompare v2.12 alpha). For CAD/CAM groups, the scan of the denture base was compared to the digital design and to the experimental model by using the same matching software. The alignment process was carried out with the help of the point pairs picking tool on the three spheres, which was created for optimization of the registration by minimizing the error distance existing between the two registered objects' surfaces. Then the mean and standard deviation were calculated by measuring the distances existing between the various points on each denture base's surface and its corresponding experimental model. Also, a color‐coded visualization map was created to express the result of comparison.

### 2.5 Statistical analysis

All data were statistically analyzed with one‐way analysis of ANOVA and the least significant difference multiple comparisons post hoc test using SPSS software (version 22). *p* Values less than .05 were considered statistically significant.

### 2.6 Ethics statement

Please note that this research only involves the use of materials. No Ethical Approval nor Informed Consent forms were required for this study.

## 3 RESULTS

**Figure 2:**
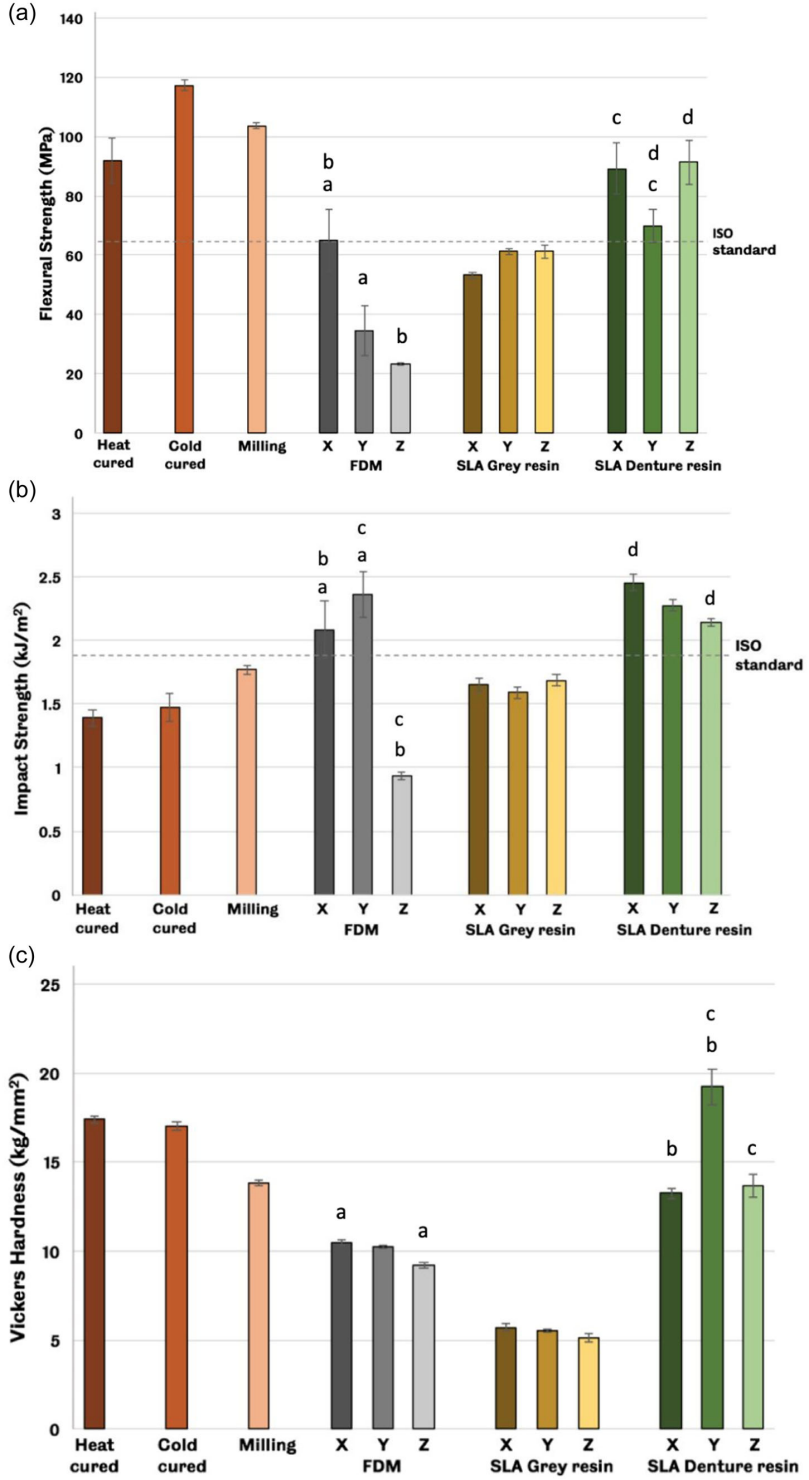
Mean values of (a) flexural strength results where the cold cure group showed the highest mean value, while the lowest mean value was in the Z FDM group (*n* = 5), (b) impact strength results where the X SLA denture resin group showed the highest mean value while the lowest mean value was in the Z FDM group (*n* = 10), and (c) Vickers hardness results where the Y SLA denture resin group showed the highest Vickers hardness values while SLA gray resin groups exhibited the lowest Vickers hardness values (*n* = 10). Error bars represent the standard error of the mean (SEM). Matching lowercase letters denote significant differences between groups (least significant difference [LSD] post hoc test, *p* < .05). FDM, fused deposition modeling; SEM, scanning electron microscopy; SLA, stereolithography.

Cold‐cured samples showed the highest mean value of flexural strength, while the lowest mean value was in the Z FDM group, as shown in Figure 2a. For impact strength, the X SLA denture resin group showed the highest mean value, while the lowest mean value was in the Z FDM group, as shown in Figure 2b. The Y SLA denture resin group showed the highest Vickers hardness values, while the SLA gray resin groups exhibited the lowest Vickers hardness values, as shown in Figure 2c.

**Figure 3:**
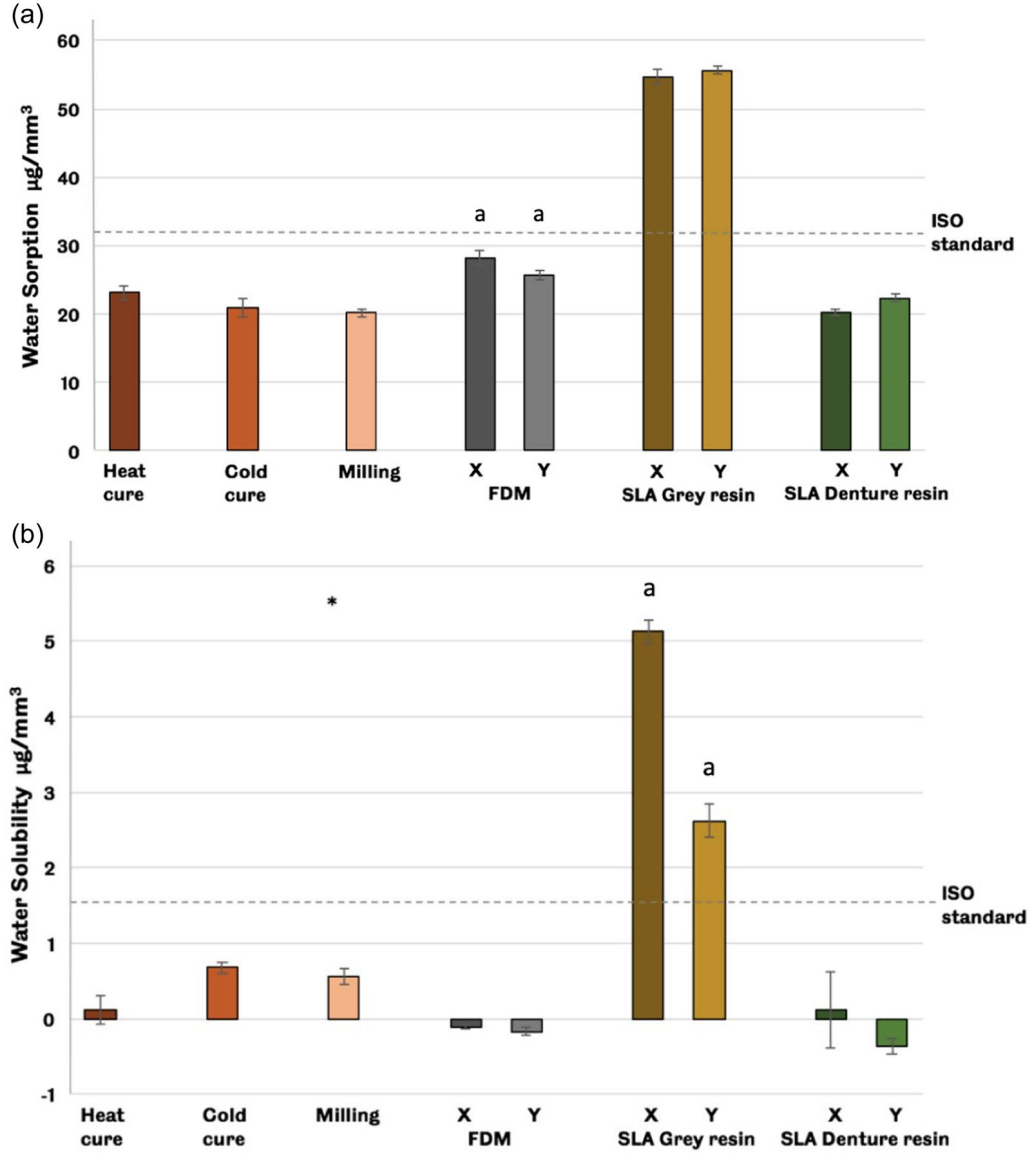
Mean values of (a) water sorption results and (b) solubility results. In both results, all experimental groups except SLA gray resin achieved ISO specifications. * (5.49) is the value of one sample of the milling group. Error bars represent standard errors. Matching lowercase letters denote significant differences between groups (least significant difference [LSD] post hoc test, *p* < .05). SLA, stereolithography.

The highest mean value of water sorption can be seen in SLA (gray resin) groups, while the lowest mean value among the groups is shown in both the milling group and SLA (X denture resin), as shown in Figure 3a. For solubility, the SLA (X gray resin) group showed the highest mean value; however, the SLA (Y denture resin) group showed the lowest mean value among groups, as shown in Figure 3b.

**Figure 4:**
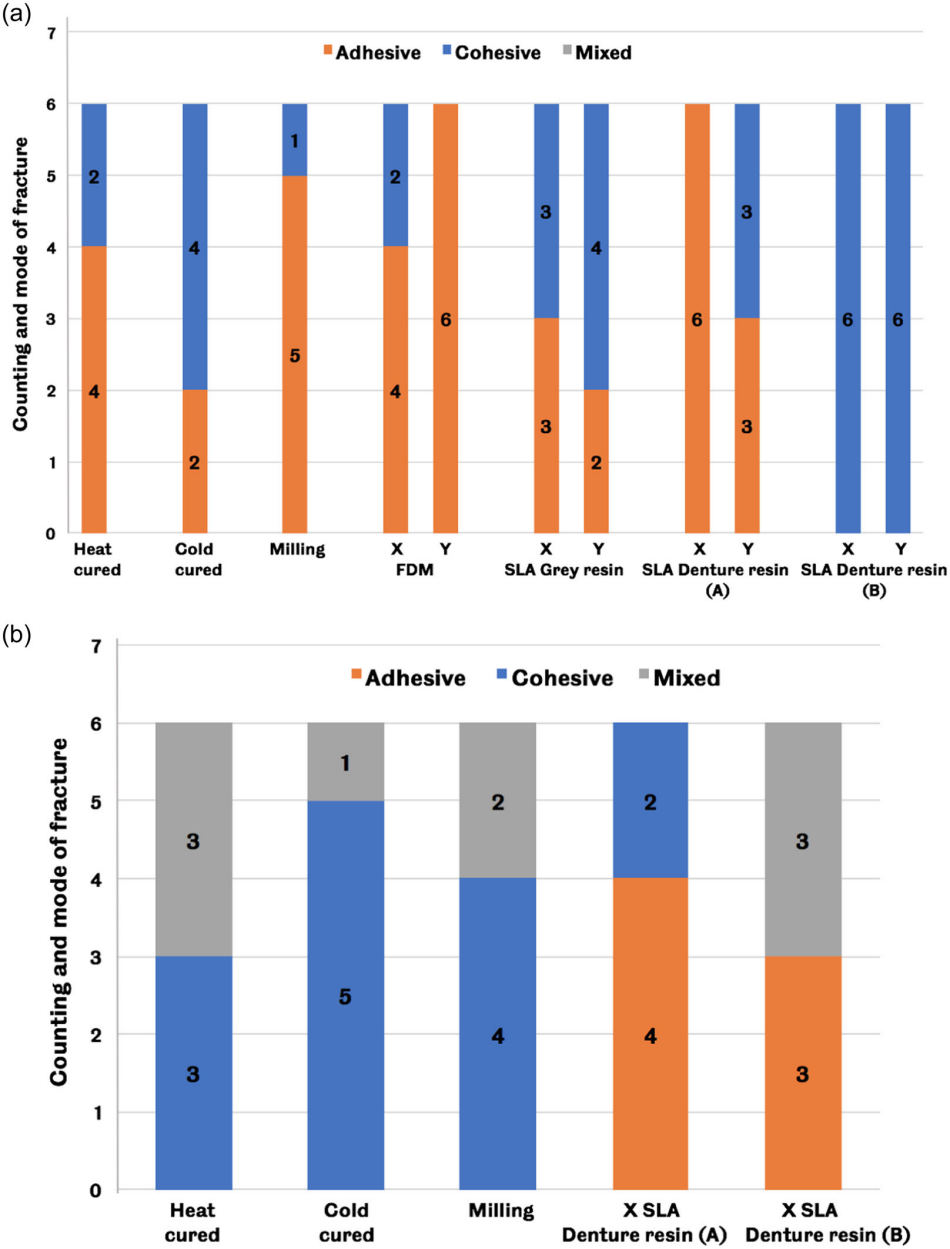
Counting and mode of fracture (adhesive, cohesive, or mixed) of tooth bonding samples for (a) the first cycle, A: with plastic teeth, B: with printed teeth, and (b) the second cycle, A: with uncured denture base resin, B: with self‐cure resin. Four out of six samples should show cohesive or mixed fracture to achieve ISO requirements. SLA, stereolithography.

Results of the tooth bonding test are presented in 2 cycles; the first cycle is ISO‐based only (with no adjustment on the prefabricated teeth), while the second cycle is ISO‐based and practice‐based (with mechanical and chemical adjustment on the prefabricated teeth). Figure 4 shows the result of counting and mode of fracture, which is used to determine the pass or fail of the samples according to ISO specifications (International Organization for Standardization, 2017) for the first cycle and the second cycle.

**Figure 5:**
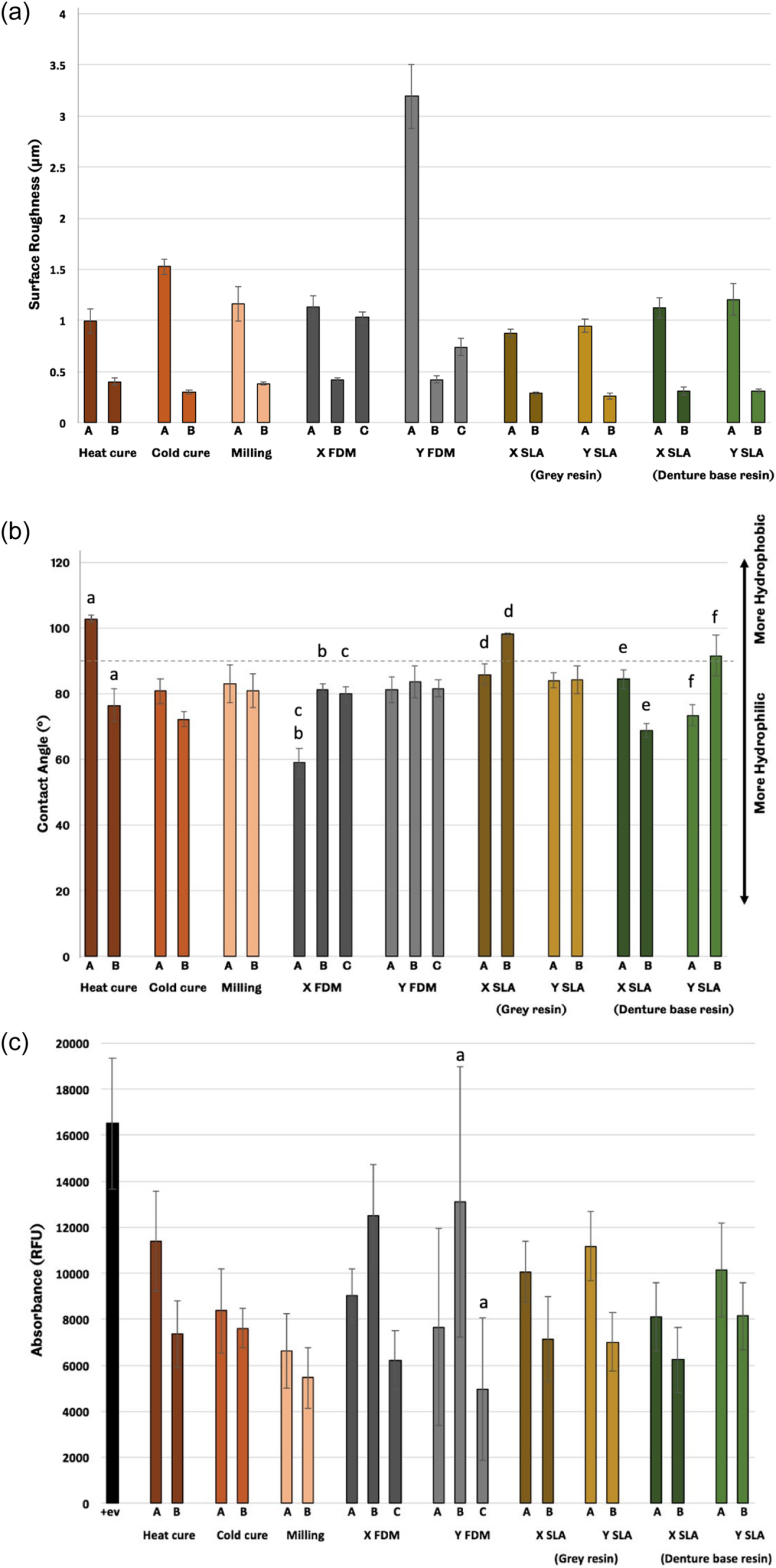
Mean values of (a) surface roughness results showed statically significant difference (*p* = .0) between different surface conditions (A & B) of all groups except FDM samples which showed no significant difference, (b) contact angle results showed that all groups except (heat cured and X FDM) show no statistically significant difference between different surface conditions (A&B&C), and (c) *Candida* viability results where the metabolic activity of *Candida* on all groups; except FDM, shows that standardized finishing samples (B) of all groups display lower metabolic activity of Candida than as processed samples (A). A: as processed, B: standardized finishing, C: acetone vapor finishing. Error bars represent standard errors. Matching lowercase letters denote significant differences between groups (least significant difference [LSD] post hoc test, *p* < .05). FDM, fused deposition modeling; SLA, stereolithography.

**Figure 6:**
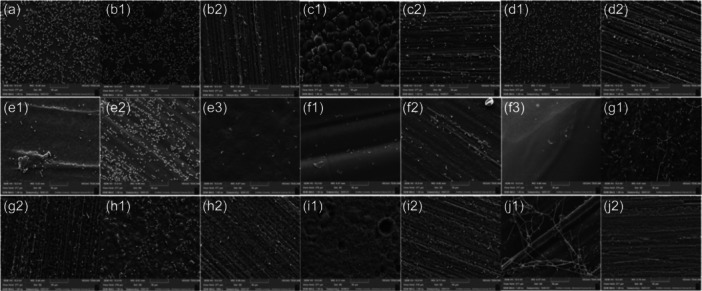
Scanning electron microscope (SEM) images (at ×1000 magnification) of *Candida* *albicans* colonization on the surface of; (a) glass disc (as a positive control), (b1) as processed heat cured sample, (b2) standardized finished heat cure sample, (c1) as processed cold cured sample, (c2) standardized finished cold cured sample, (d1) as processed milled sample, (d2) standardized finished milled sample, (e1) as processed X FDM sample, (e2) standardized finished X FDM sample, (e3) acetone vapor finishing X FDM sample, (f1) as processed Y FDM sample, (f2) standardized finished Y FDM sample, (f3) acetone vapor finishing Y FDM sample, (g1) as processed X SLA (gray resin) sample, (g2) standardized finished X SLA (gray resin) sample, (h1) as processed Y SLA (gray resin) sample, (h2) standardized finished Y SLA (gray resin) sample, (i1) as processed X SLA (denture base resin) sample, (i2) standardized finished X SLA (denture base resin) sample, (j1) as processed Y SLA (denture base resin) sample, and (j2) standardized finished Y SLA (denture base resin) sample. FDM, fused deposition modeling; SLA, stereolithography.

The surface roughness results of different surface conditions of tested groups are shown in Figure 5a. There is a statically significant difference (*p* = .0) between different surface conditions (A & B) of all groups except FDM samples which showed no significant difference (*p* = .496) between A & C of X FDM group and (*p* = .031) between B & C of Y FDM group. The mean values of contact angle measurements of each group against distilled water are shown in Figure 5b. All groups except (heat cured and X FDM) show no statistically significant difference between different surface conditions (A&B&C). The heat‐cured group shows a significant difference (*p* = .0) between different surface conditions (A & B), while the significant difference is found in X FDM when comparing A to B &C while there is no significant difference between B &C. Metabolic activity of *C. albicans* on all groups; except FDM, cell viability agent (Presto Blue) shows that standardized finishing samples (B) of all groups display lower metabolic activity of *C. albicans* than processed samples (A), as shown in Figure 5c. However, FDM samples (both orientations, X & Y) show that the standardized finishing samples (B) have higher metabolic activity than processed samples (A), followed by the acetone vapor finishing samples (C). SEM images are shown in Figure 6.

**Figure 7:**
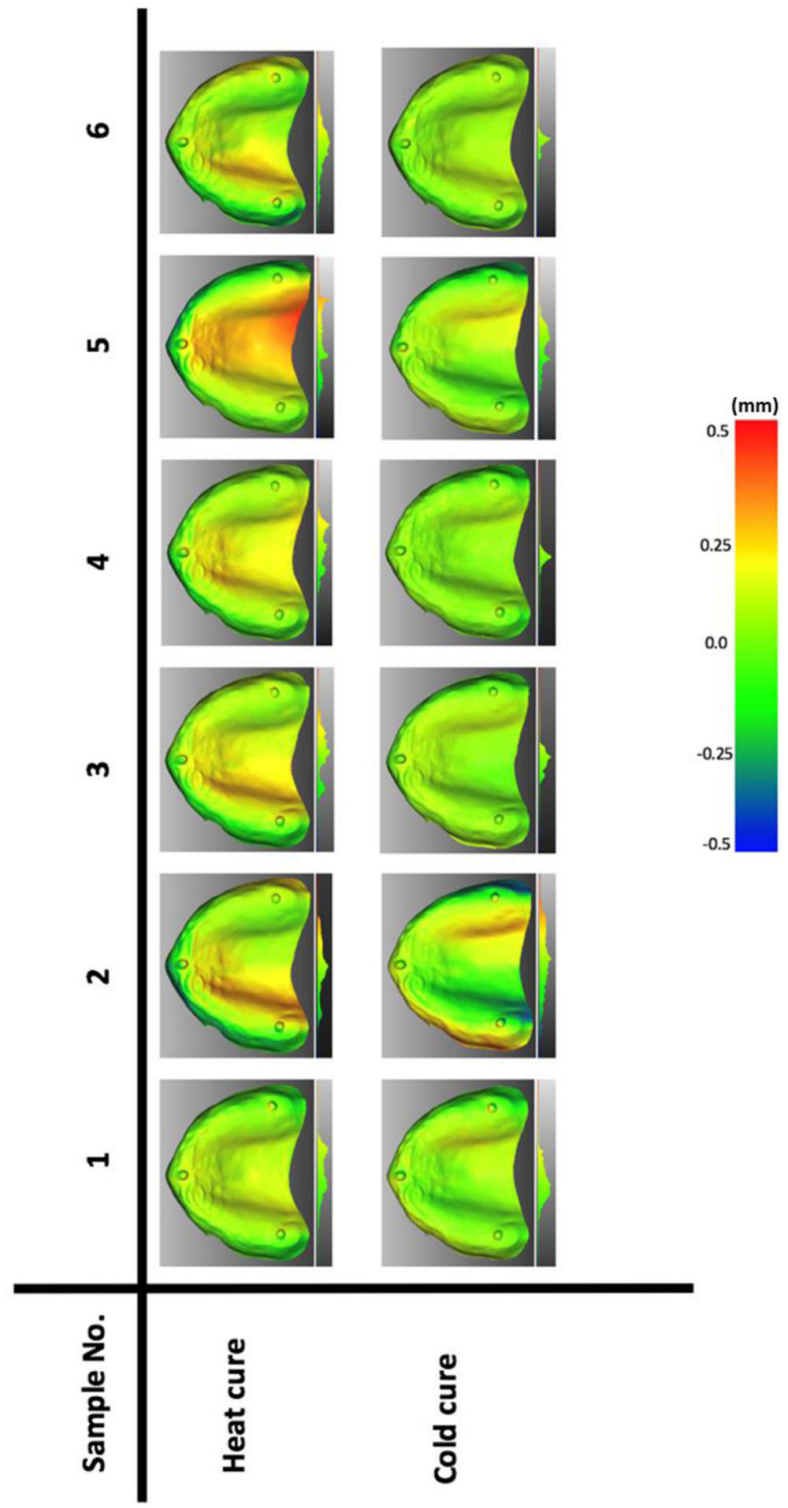
Color‐coded maps for heat and cold cure samples.

**Figure 8:**
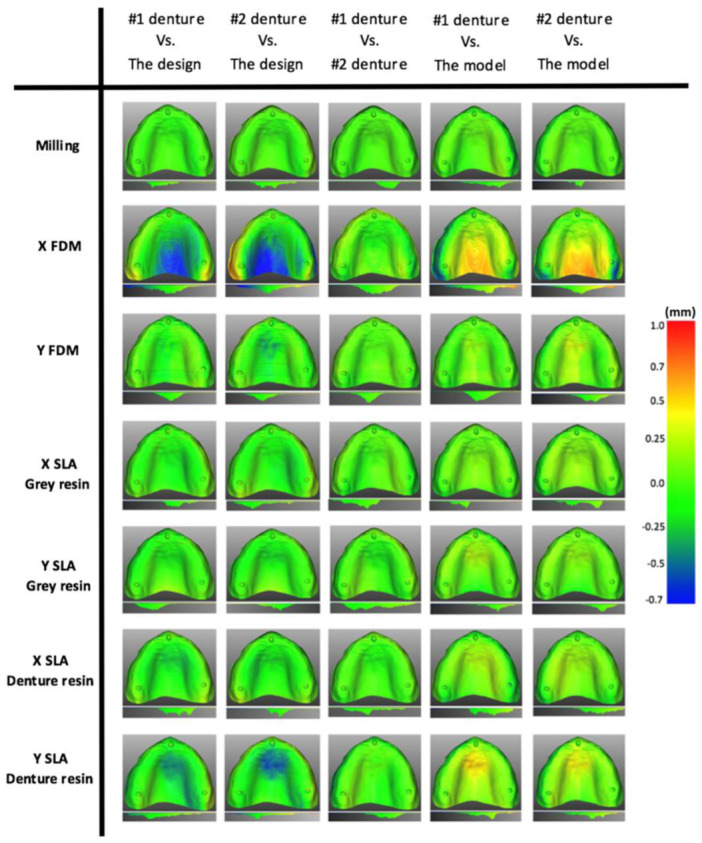
Color‐coded map of surface matching for CAD/CAM samples (the third step). Each denture base of the tested groups was compared to the design (CAD), another denture base, and the model. CAM, computer‐aided manufacturing; FDM, fused deposition modeling; SLA, stereolithography.

Each conventional denture was compared to its corresponding model, and a color map of heat and cold cure samples is shown in Figure 7, where cold cure samples showed fewer discrepancies of fit than heat cure samples. The scale of 0.5 to −0.5 in the color map was set for conventional samples, while the scale of 1 to −0.7 was set for CAD/CAM samples; the scale value is determined based on the worst result (the maximum distance) in the matching analysis. The process of evaluating the accuracy of fit for CAD/CAM groups passes through three steps, as shown in Supporting Information S1: Figure 3 in the appendix. The results of the first and the second steps, which are shared between all groups (milling, FDM, and SLA), are shown in Supporting Information S1: Figure 4 in the appendix, while the results of the third step for each group are demonstrated in Figure 8. A summary highlighting key statistical parameters for all the properties studied above is presented in Table 2.

**Table 2:** *F*‐test by ANOVA to show the statistical differences between examined groups.

	Flexural strength	Impact strength	Vickers hardness	Water sorption	Water solubility	Surface roughness	Contact angle	Viability test
*F‐*test	22.761	21.662	157.159	278.134	21.468	43.338	6.687	1.517
*p* Value	.00	.00	.00	.00	.00	.00	.00	.126

## 4 DISCUSSION

According to Deng et al. (2018), it has not been possible to identify a 3D printing process that has totally achieved the clinical demand criteria for complete dentures, including mechanical properties, biocompatibility, and esthetic parameters. This study has compared conventional and additive‐based manufacturing techniques and key demand criteria for denture base performance to investigate the feasibility of using 3D‐printed PMMA filament as clinically relevant denture base material. The results of this study led to the rejection of the null hypothesis and confirmed that variations in manufacturing techniques do occur.

Flexural strength results showed variation across the sample groups, with many of the 3D‐printed samples performing below the ISO requirements. The FDM group showed greater variation, with the “X” printed samples exceeding the ISO standard, and the “Y & Z” samples below the standard. The findings of this study agree with previously published work demonstrating that printing orientation is a significant manufacturing parameter that impacts the mechanical properties of 3D‐printed objects (Chantarapanich et al., 2013; Dizon et al., 2018; Mohamed et al., 2015). Impact strength results showed that only two of the FDM subgroups (X & Y) and the SLA denture resin group passed the ISO specifications. However, conventional groups failed to achieve ISO specifications in terms of impact strength, and this finding agrees with previously published literate (Aguirre et al., 2020; Al‐Dwairi et al., 2020) in which it was concluded that conventional resins showed lower impact strength values in compared to CAD/CAM resins. The FDM group demonstrated a significant change with printing orientation, with the “Z” samples (where the layers were in alignment with the direction of impact force) performing significantly worse (more than 50% decrease from the upright samples), meaning that knowledge of the likely applied force needs to be considered in a design and printing orientation. The hardness test showed that the Y SLA denture resin group produced the hardest samples, followed by heat and cold cure groups, and all samples offered a shiny appearance after the polishing procedure. The degree of surface smoothing is crucial in decreasing the accumulation of microorganisms and retaining undesirable particles, thus enhancing the cleaning procedure of removable dental prostheses (Al‐Rifaiy, 2010).

Water sorption results presented that all groups achieved ISO specifications except both subgroups (X&Y) of the SLA gray resin group. The FDM group and SLA denture resin group showed no significant difference between the two subgroups (printing orientations groups, X & Y). This means there are no restrictions on printing the denture base with a specific position. Similar to the water sorption results, all groups passed the ISO specifications for the solubility test except the SLA gray group in both orientations. The cold cure group showed higher solubility values than the heat cure, while the milling group performed within the border of the ISO requirements. This finding agrees with previously published research in which heat cure resin was processed under high temperature for a long duration and thus resulted in low residual monomer concentration and, therefore, low solubility rates (Figuerôa et al., 2018; Miettinen & Vallittu, 1997; Tuna et al., 2008).

In the tooth bonding study, the count and mode of fracture of the first cycle were investigated, and it was found that adhesive failure occurred in most of the groups. Only cold cure, Y SLA gray resin, and SLA denture base resin (B) groups passed the ISO requirements for the tooth bonding test. The present method of the first cycle is problematic since the variation in monomeric contacts between the groups possibly influences the results. To avoid this problem and to standardize on all tested groups, the second cycle was created where the fitting surface of the prefabricated teeth was roughened and painted with methyl methacrylate monomer. All groups of the second cycle except (A)&(B) X SLA denture base groups pass the ISO specifications in terms of counting and mode of fracture of tooth bonding test. Tooth bonding failure is a common scenario in clinical practice (Bhochhibhoya et al., 2016) and to minimize this failure, many attempts, such as mechanical modification and chemical treatment, have been investigated (Cardash et al., 1990; Chung et al., 2008; Fletcher et al., 1985; Spratley, 1987). The finding of the second cycle concurred with the literature, where the method of roughening the fitting surface of the prefabricated teeth to remove the glazed layer and applying methyl methacrylate monomer enhanced the results of fracture mode to be within the ISO standards specifications. According to Bhochhibhoya et al. (2016), the maximum masticatory force created by complete denture individuals is about 90 N, and based on the present results (Supporting Information S1: Figures 1 and 2, Appendix), the shear bond strength of most groups is higher than the required force for masticatory function. Therefore, the bond strength of the tested groups would be clinically acceptable. Scientific literature has no published studies evaluating the tooth bonding strength of milling and 3D printing techniques in accordance with ISO standards. Thus, the finding of this study has added effective/valuable knowledge in terms of evaluating the bonding behavior of the tooth to the denture base fabricated by milling and 3D printing techniques.

For the microbiological characterization, the effects of surface roughness on *C. albicans* adhesion and viability were assessed. The samples of each group were distributed according to their surface morphology: (i) “as processed” to mimic the fitting surface of the denture, (ii) standardized finishing (which was performed to make the surface of the sample smooth and then assess the effectiveness of the surface roughness on the *Candida* adhesion), and (iii) acetone vapor finishing (only for FDM group) which was performed by using acetone vapor for finishing the samples' surfaces. This latter technique is known as vapor smoothing and has been introduced by Stratasys Inc., where the chemical vapors react with the outer layers of FDM parts (Chohan et al., 2017). The surface roughness values, Figure 5a, of all denture‐base materials, are higher than the threshold (0.2 μm) defined by Bollenl et al. (1997). This finding has a significant relation to the amount of *Candida* adherence where the viability analysis of *Candida* shows that more *Candida* cells adhere to rough surfaces than smooth surfaces, as observed previously (Murat et al., 2019; Radford et al., 1998; Verran & Maryan, 1997). We also measured the sample's contact angle, which is used to determine the hydrophobicity of the sample surface and its possible effect on *Candida* adherence (Kim et al., 2019; Lazarin et al., 2013; da Silva et al., 2015; Teughels et al., 2006; Zamperini et al., 2010). The highest roughness result among the tested groups was found in processed samples of Y orientation of the FDM group. This agreed with (Agarwala et al., 1996; Weeren et al., 1995), who concluded that objects processed by FDM show low surface finish, which is generated due to curve approximation or chordal error and stair‐stepping appearance; these terminologies explain the outer outline of the deposited layer. Furthermore, the SEM images show a higher percentage of *Candida* adhering to samples without final surface treatment for all groups.

Based on the presented results of accuracy of fit, cold cure dentures would show more accuracy than heat cure ones, and this finding is concurrent with literature: specifically, studies have concluded that autopolymerising resins show better accuracy of fit performance than other resins used for conventional fabrication of dentures (Al Elsheikh & Abdel‐Hakim, 1995; Craig et al., 2004). Most cold cure samples show a green color in the color map, as shown in Figure 7, which indicates fewer discrepancies between the denture and its corresponding cast. The evaluation of the accuracy of fit for CAD/CAM groups undergoes multiple comparisons, as shown in Supporting Information S1: Figure 3 in the appendix, and these comparisons are divided into three steps. The comparison in the first and second steps displayed almost identical results, as shown in Supporting Information S1: Figure 4 in the appendix, and this indicates that the process of the scanning and the denture designing (CAD) provides highly accurate outcomes (no faults associated with these procedures). The third step of comparison contains a comparison between the CAD/CAM dentures and the denture design (CAD), all groups show fewer discrepancies except the X FDM group, which means that using the FDM technique to fabricate a denture with X orientation is unable to provide a denture that highly accurate to the denture design (CAD) file. All the previous multiple comparisons were performed to answer the question of whether the error happened is it before are after milling or printing the denture. From the previous illustration of all the steps in the CAD/CAM workflow analysis, we can say that the misfit of the denture is more likely due to the process of manufacturing (printing errors).

Although successful in different ways, this study shows several limitations; the common and shared limitation is related to the PMMA filament, which is not approved to be used intraorally and is of unesthetic appearance, being transparent in color. Another limitation is related to the resolution of printing and the layer thickness which is not consistent among FDM and SLA techniques. The 0.1 mm layer thickness has been determined to be a layer thickness in this study and FDM samples were manufactured within this thickness. The Form 2 denture base resin was released in the United Kingdom market recently, and this resin is designed to be printed with one specific layer thickness, which is 0.05 mm. So, the FDM samples were printed with 0.1 mm and the SLA samples were printed with 0.05 mm. Moreover, several parameters that control the printing process, such as line width, print speed, and layer height, were not evaluated in this investigation. The inserted values of these parameters might impact the mechanical properties of 3D‐printed samples. One study has advocated that the mechanical features of 3D‐printed products are affected by processing parameters such as layer height and width (Mohamed et al., 2015). Within the tooth bonding test, the nature of the applied load is different where in the oral environment, the masticatory load is dynamic during the function, while in the performed test, the load was static. This in vitro study is carried out within laboratory conditions where clinical aspects have not been assessed over a long time. The 3D‐printed denture has not been used intraorally for enough time to evaluate the resistance of this technique against intraoral factors such as the masticatory force and the wet environment. Therefore, it is hard to draw a conclusion regarding the materials and the techniques.

## 5 CONCLUSION

We have demonstrated that FDM provides denture base constructs with good mechanical performance and stability in a wet environment; however, these constructs do not comply with ISO specifications for tooth bonding and show poor accuracy of fit as well as poor surface finish (which leads to undesirable high *Candida* adhesion). In essence, this work has shown that the FDM technique is not yet ready to be used in the fabrication of a denture base for clinical use and that there is a need for current ISO testing regimes for denture base polymers to be revised so they can meet the demands required by state‐of‐the‐art dental manufacturing techniques.

## AUTHOR CONTRIBUTIONS

*Conceptualization*: Ilida Ortega Asencio, Christopher W. Stokes, and Duncan Wood. *Funding acquisition*: Khalid K. Alanazi, Christopher W. Stokes, and Ilida Ortega Asencio. *Investigation*: Khalid K. Alanazi, Christopher W. Stokes, Ilida Ortega Asencio, Duncan Wood, and Joanna Shepherd. *Methodology*: Khalid K. Alanazi, Christopher W. Stokes, Ilida Ortega Asencio, Duncan Wood, and Joanna Shepherd. *Resources*: Christopher W. Stokes, Ilida Ortega Asencio, Duncan Wood, and Joanna Shepherd. *Supervision*: Christopher W. Stokes, Ilida Ortega Asencio, Duncan Wood, and Joanna Shepherd. *Writing—original draft*: Khalid K. Alanazi and Ilida Ortega Asencio. *Writing—review and editing*: Khalid K. Alanazi, Christopher W. Stokes, Ilida Ortega Asencio, Duncan Wood, and Joanna Shepherd.

## CONFLICT OF INTEREST STATEMENT

The authors declare no conflict of interest.

## Supporting information

Supporting information.

## Data Availability

Data that support the findings of this study are available on request from the corresponding author. The data are not publicly available due to privacy.
