# Supplementary material for: Assessing the suitability of fused deposition modeling to produce acrylic removable denture bases
Source: Clin Exp Dent Res. 2024 May 27;10(3):e880. doi: 10.1002/cre2.880 (PMC11128773; doi:10.1002/cre2.880)
Supplement: Supplementary file 1 — Supporting information. [file CRE2-10-e880-s001.docx]

**Appendix**


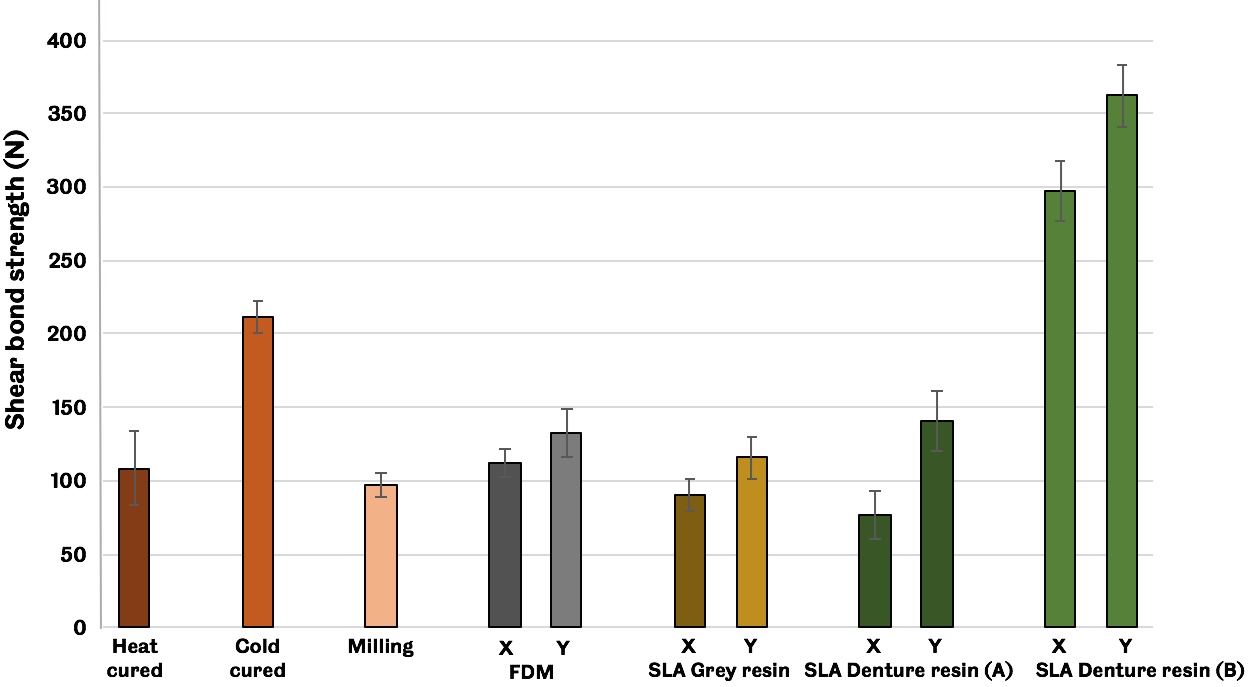


**Figure 1.** Mean values of shear bonding strength for all groups of the first cycle. A: with plastic teeth, B: with printed teeth. Error bars represents standard errors.


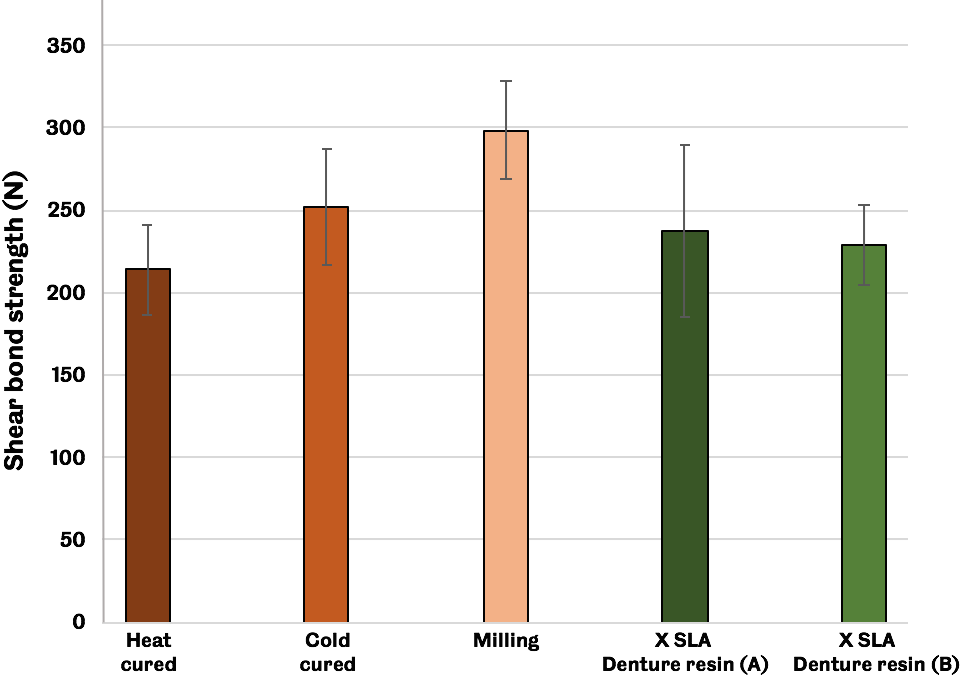


**Figure 2.** Mean values of shear bonding strength for all groups of the second cycle. A: with uncured denture base resin, B: with self-cure resin. Error bars represent standard errors.


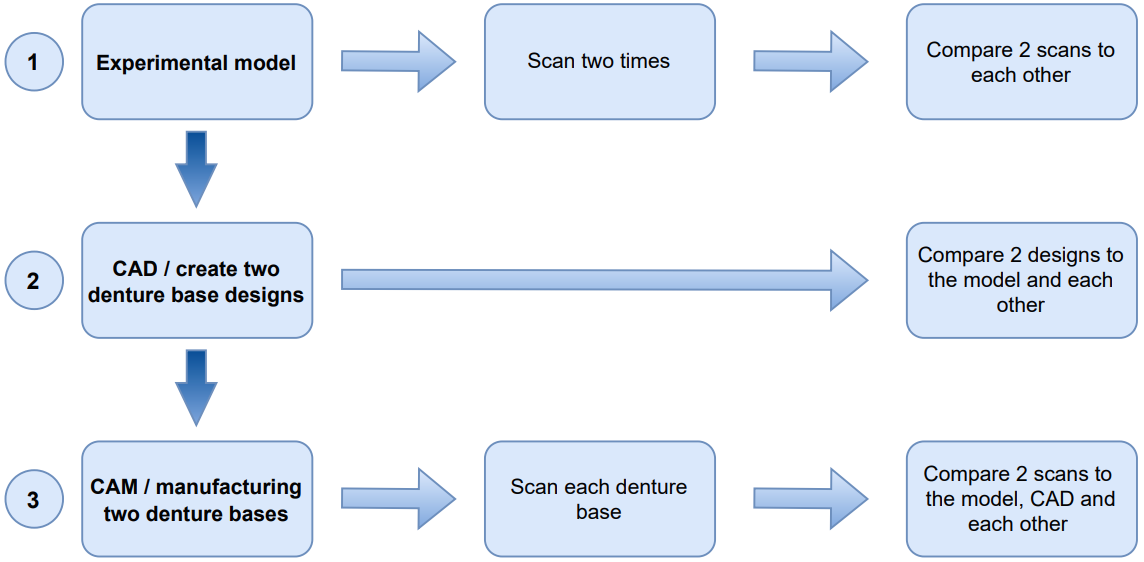


**Figure 3.** Chart illustrates the three steps in evaluating the accuracy of fit of CAD/CAM groups. In the first step, the experimental model is scanned two times then these 2 scans compared to each other. In the second step, one of the experimental model scans is used to create two denture base designs (CAD), then these designs are compared to the scan and to each other. In the third step, one of the denture designs is used to manufacture 2 denture bases through CAD/CAM techniques, then each denture base is scanned and compared to the model, CAD, and each other.


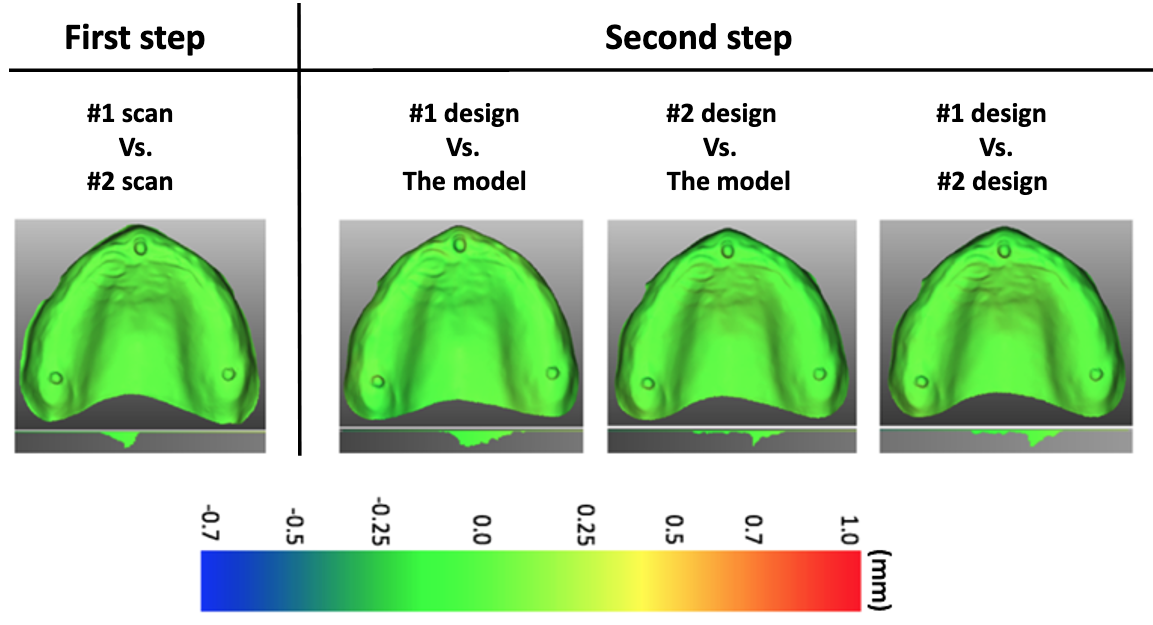


**Figure 4.** Colour coded map of the first and second steps.
